# National survey of Dutch emergency physicians on pharmacological sedation practices for extreme agitation

**DOI:** 10.1016/j.toxrep.2026.102246

**Published:** 2026-03-28

**Authors:** J.J.J. Ouwerkerk, N. Kraaijvanger, N.J.A. van der Wee, F.M.J. Gresnigt

**Affiliations:** a Emergency Department, Leiden University Medical Centre, Leiden, the Netherlands; b Department of Psychiatry and Psychology, Leiden University Medical Centre, Leiden, the Netherlands; c Emergency Department, OLVG, Amsterdam, the Netherlands; d National Poison Information Centre, University Medical Centre Utrecht, Utrecht, the Netherlands

**Keywords:** Acute behavioural disturbance, Extreme agitation, Sedation, Tranquilization, Survey

## Abstract

**Background and importance:**

Extreme agitation in the emergency department (ED) is a high-risk scenario requiring rapid, safe sedation. International evidence and guidance on first line and rescue agents are variable, however current practice has not been systematically described.

**Objectives:**

To characterise pharmacological sedation practices reported by Dutch emergency physicians (EPs) for the management of extreme agitation in the ED, including agent selection, dosing, determinants of choice, perceived performance, and rescue strategies,

**Methodology:**

National cross-sectional web-based survey among Dutch board-certified EPs (and EPs in training), was distributed to all Dutch Society of Emergency Physicians (DSEP) members through a newsletter, at the yearly DSEP conferences, to colleagues from prior collaborations, and via LinkedIn.

**Results:**

Of the 679 eligible board-certified EPs, 293 responded (43.2%); after excluding 42 incomplete responses, 251 were analysed (37.0%). Among this group, 39.0% reported that prehospital midazolam sedation, administered by ambulance services, was often insufficient upon patient arrival in the ED. Without available intravenous (IV) access, the initial choices of sedative most often were midazolam (34.7%), droperidol (23.1%), the droperidol–midazolam combination (14.3%), and esketamine (10.4%), with intramuscular (IM) being the preferred route in these situations; The median IM doses were 10 mg for droperidol and midazolam, 5 mg each for the droperidol–midazolam combination, and 150 mg for esketamine. With IV access, the same ranking applied, with IV being the preferred route, although a small proportion of respondents still opted for alternative routes such as IM and intranasal (IN). The most cited reasons for agent choice, were the DSEP pocket card on sedation for behavioural disturbance (43.0%), perceived effectiveness (37.8%), and residency training (30.3%). When asked about personal experience with sedatives, droperidol and esketamine were frequently described as effective and rapid, whereas midazolam was more often linked to side effects. When initial sedation failed, 85.7% escalated the initial sedative to a certain dosage before switching; esketamine (32.7%) and propofol (27.5%) were the most common rescue medications.

**Conclusion:**

Dutch EPs primarily use a recommended set of sedatives based on national guidelines, but their approach to managing extreme agitation varies within that framework. Emergency physicians reported midazolam as the most frequently used first-line agent, although respondents generally rated droperidol as more effective and better tolerated. Given these perceptions and the available evidence supporting droperidol’s efficacy and safety, it may be reasonable to reconsider the prominent role of midazolam, particularly since prehospital midazolam sedation was often judged to be insufficient.

## Highlights

● Prehospital midazolam often judged insufficient.
● Midazolam most used, yet linked to more side effects.
● Droperidol and esketamine rated faster and better tolerated.

## 1 Introduction

Extreme agitation, historically referred to as “excited delirium” and additionally described as “hyperactive delirium with severe agitation”, in the prehospital and emergency department (ED) setting, represents a high-risk clinical scenario that demands immediate and effective intervention [1]. This condition, often arising from intoxication, withdrawal, psychosis, or mania, is associated with a substantial risk of severe complications, including hyperthermia, rhabdomyolysis, cardiac arrhythmias, and death [1], [2]. It also poses a significant threat to the safety of healthcare personnel [3]. The true incidence of extreme agitation is difficult to estimate, due to inconsistent definitions, scarce incidence data and its variation by setting and underlying aetiology. In a large observational study at an urban United States ED, 2.6% of ED patients showed significant agitation, with most requiring physical restraint (84%) and intramuscular (IM) sedation administration (72%) [4]. Additionally, a German prehospital study reported signs of agitation or violence in 3.2% of patients transported by emergency medical services [5].

The primary therapeutic goal is to achieve rapid behavioural control through pharmacological sedation, thereby preventing complications and enabling safe diagnostic and therapeutic procedures. The ideal sedative acts quickly, has no side-effects, requires only a single administration, has a relative short duration of action, and can preferably be administered through multiple routes. When patients are cooperative and able to take medication, the oral route may be an appropriate and guideline-supported option according the American Association for Emergency Psychiatry [6], [7]. However, in patients with extreme agitation this is often not feasible. In such cases, intramuscular (IM) administration is generally preferred as intravenous (IV) access is often difficult or unsafe to obtain and IM protocols have been associated with faster control of agitation and fewer staff injuries [8]. Although multiple sedatives have been studied for the management of extreme agitation, there is ongoing debate about which agent offers the best balance of efficacy and safety [9], [10]. Midazolam, one of the most commonly used sedatives worldwide, is valued for its rapid onset and considered safety, but is frequently associated with respiratory depression and airway obstruction [9], [11], [12]. Droperidol has demonstrated reliable and stable sedation [11], [12], [13]; however, concerns about QTc prolongation, led to a substantial decline in its use following the Food and Drugs Administration (FDA) black box warning in 2001 [14], [15]. Recent evidence, however, suggests that the actual risk of QTc-related complications is minimal, and droperidol is gradually being reintroduced into clinical practice internationally [16], [17], [18], [19]. Furthermore, ketamine is increasingly recognised as an effective sedative with rapid onset for the management of severe agitation. Most available evidence relates to racemic ketamine, while data on esketamine for this indication remain limited. Esketamine, the S-enantiomer of ketamine, is approximately twice as potent, but comparative evidence in acute agitation is scarce. In the Netherlands, esketamine rather than racemic ketamine is only available for clinical use [20]. In prehospital settings, ketamine use has been associated with a higher incidence of intubation, which has led some authors to suggest reserving it primarily as a rescue medication rather than a first-line sedative agent [9], [21], [22], [23]. Other sedatives such as haloperidol, lorazepam, and promethazine are generally less suitable for emergency use, due to their delayed onset of action [9], [24], [25].

There is currently no universally adopted protocol for the pharmacological management of extreme agitation. At present, the European Society for Emergency Medicine (EUSEM) has not issued a clinical guideline addressing this topic. Since 2023, the Dutch Society of Emergency Physicians (DSEP) recommends midazolam, droperidol, or their combination as initial treatment (see appendix 1 for DSEP sedation pocket card for acute behavioural disturbances) [26]. In contrast, the United Kingdom-based Royal College of Emergency Medicine (RCEM) advises esketamine as the preferred initial agent [27]. In Australia, the New South Wales Health institution recommends droperidol as first-line treatment for acute severe behavioural disturbance in the ED [28]. Similarly, the American College of Emergency Physicians (ACEP) clinical policy recommends droperidol or an atypical antipsychotic, alone or in combination with midazolam, for rapid and effective management of severe agitation [29]. This illustrates that there is no international consensus on the optimal pharmacological approach to extreme agitation. Although existing guidelines are grounded in clinical experience and supported by a growing body of evidence, high-quality comparative studies remain are scarce and there is insufficient data to definitively determine which agent is the most effective and safe sedative for extreme agitation [9]. To optimize the design of future clinical trials and ensure their relevance to routine practice, it is important to first identify which sedative agents are currently being used in everyday clinical care and to understand the experiences and perspectives of the physicians administering them.

Therefore, this study aims to describe current sedation practices for extremely agitated patients in Dutch EDs, particularly following the recommendation for droperidol as first choice for IM administration in extreme agitation by DSEP since 2023 [26]. We explore which agents are purportedly most commonly used by emergency physicians (EPs), what factors influence physicians’ choice of sedative, and how they manage cases in which initial sedation fails. Additionally, by examining clinician-reported experiences with different agents, this study seeks to highlight perceived advantages and limitations of each sedative. These insights will help inform the design of future clinical trials and contribute to the development of evidence-based, context-specific, clinically relevant sedation strategies.

## 2 Methodology

The survey was available online between May and October 2025. The aim of the study was to examine current pharmacological sedation practices for extreme agitation among Dutch EPs.

A web-based questionnaire was developed using Castor Electronic Data Capture (Castor EDC) [30], based on a review of the available literature and expert opinion (see appendix 2 for the survey). The survey assessed EPs’ characteristics, sedative strategies for extreme agitation, commonly used sedatives, and clinicians’ experiences with different agents. It consisted predominantly of multiple-choice questions, with optional free-text fields to capture additional comments or clarifications. Reasons for selecting a specific sedative agent were provided as predefined answer options in the questionnaire (a priori), based on a literature review and expert opinion; participants could additionally provide free-text responses if desired. Appreciation was rated for each sedative the respondent had previously used on a 5-point Likert-type scale ranging from very negative to very positive. The survey was targeted at physicians who were board-certified in emergency medicine and those who were currently enrolled in a recognized emergency medicine training program in the Netherlands. In the Netherlands, not all hospitals are employing EPs. Furthermore, some hospitals have EP coverage only during part of the rotation schedule. Exclusion criteria included incomplete responses and current employment outside of the Netherlands.

To facilitate national distribution, all hospitals in the Netherlands with an ED staffed by EPs were contacted by telephone, requesting for contact details of a staff member responsible for internal research coordination. These contact persons were invited to distribute the survey to eligible EPs and emergency residents within their departments. Two follow-up reminder emails were sent to all contacts, with additional reminders targeted at non-responding hospitals. Additionally, the survey was distributed to all DSEP members through a newsletter, at the yearly DSEP conferences, colleagues from prior collaborations, and via LinkedIn.

Data collection was carried out through the Castor platform. All data were exported to RStudio (version 16.0) [31] for processing, and descriptive analysis. Geographic classification (urban, intermediate or rural) of the hospital was based on the level of urbanization defined by the Dutch national statistics agency (CBS) using the StatLine database, which categorises municipalities by address density [32]. Hospitals were also classified by trauma centre level. Level 1 trauma centres provide comprehensive trauma care with 24/7 specialised resources; level 2 centres provide advanced trauma care with more limited specialised services; and level 3 centres provide initial trauma stabilisation with referral to higher-level centres when needed. Analyses included frequency distributions, response proportions, and cross-tabulations of relevant variables. No statistical hypothesis testing was performed, as this was a descriptive study. Dosages were analysed per administration route. IM, IV, and IN doses were each allocated to their respective route categories. When a combined single administration was reported (e.g., simultaneous IM and IN), each dose was analysed separately and assigned to the appropriate route category. Only the most reported used sedatives and sedative combinations were included in the analysis and presented in this study; Sedatives or combinations of sedatives occurring in < 2% of responses were grouped as “others” and not shown individually. The same rule was applied in the appendix, with limited exceptions (e.g. esketamine in trainees) to maintain consistency with the main tables. Data from EPs in training were analysed separately, as their clinical exposure and independent decision-making experience differ substantially from board-certified EPs; combining both groups could disproportionately weight less experienced perspectives in the analysis. Board-certified EPs refer to EPs who have completed their specialist training and are registered as EP according to the DSEP. During the preparation of this work, the author(s) used ChatGPT (OpenAI) to assist in drafting and refining RStudio syntax for data analysis; ChatGPT was not used for drafting the manuscript text. All generated code was critically reviewed, tested, and adapted as necessary by the author(s), who take full responsibility for the final analyses and the content of the published article. Full survey content and statistical code are available from the corresponding author upon request.

This study adhered to the Strengthening the Reporting of Observational Studies in Epidemiology (STROBE) guidelines and the Reporting of Studies Conducted Using Observational Routinely-Collected Health Data (RECORD) extension [33], [34]. The study was reviewed by the LUMC Institutional Review Board and classified as exempt from formal ethical review under Dutch law.

## 3 Results

According to the Dutch Society of Emergency Physicians (DSEP) [35], a total of 679 board-certified EPs were working in the Netherlands during the survey period. Of these, 293 (43.2%) responded. After excluding 42 responses (6.2%) due to missing or incomplete data, 251 responses (37.0%) were included in the final analysis. Unless otherwise specified, the results section presents the characteristics and responses of board-certified EPs; full data from EPs in training are provided in appendix 3 till 8. Regarding national coverage, the Netherlands has 59 hospitals with one or more ED locations, employing EPs; from all 59 hospitals (100%) at least one response was provided to the survey.

### 3.1 Baseline characteristics

Of the physicians included in the final analysis, 251 were board-certified in emergency medicine. For the board-certified EPs, the median duration of working experience in the ED was 14.0 years (IQR 10.0–18.0). Regarding hospital characteristics, 220 (87.6%) respondents worked in urban centres, and 25 physicians (10.0%) were employed at an academic medical centre.

### 3.2 Sedative choice

When specifically examining prehospital sedation, 45.0% of EPs reported that patients receiving prehospital sedation were generally adequately sedated upon ED arrival, while 39.0% reported that the level of sedation upon ED arrival was insufficient. Regarding initial pharmacological choices in the ED when no IV access was available, the IM route was preferred over the intranasal (IN) route. In patients with no IV access, midazolam was the most reported used agent (34.7%), followed by droperidol (23.1%), a combination of midazolam and droperidol (14.3%), and esketamine (10.4%) (Table 2). In patients with IV access, the IV route was the preferred option, and the same order of initial sedative preference was registered, although the average initial doses tended to be lower. A small proportion of respondents reported using other sedatives or uncommon combinations, most of which involved midazolam (e.g. midazolam with lorazepam, midazolam with haloperidol, or midazolam with promethazine), as well as infrequent combinations such as lorazepam with haloperidol.

**Table 1:** Characteristics of participating EPs (board Certified) (*n* = 251).

Sex (*n*, %)	
Male	87 (34.7%)
Female	163 (64.9%)
Other	1 (0.4%)
Age (years)	
Between 20 and 29	0 (0.0%)
Between 30 and 39	93 (37.1%)
Between 40 and 49	123 (49.0%)
Between 50 and 59	33 (13.1%)
Between 60 and 69	2 (0.8%)
Duration of clinical experience	
As physician in general (years, SD)	15.0 (12.0–20.0)
As physician working in the ED (years, SD)	14.0 (10.0–18.0)
Hospital characteristics	
24/7 staffing with EPs	192 (76.5%)
Geographic classification, *n* (%)	
Urban	220 (87.6%)
Intermediate	24 (9.6%)
Rural	7 (2.8%)
Trauma level, *n* (%)	
Level 1	54 (21.5%)
Level 2	170 (67.7%)
Level 3	27 (10.8%)
Academic status	25 (10.0%)

SD = Standard deviation; EP = Emergency Physician; ED = Emergency Department

**Table 2:** Characteristics of initial pharmacological sedation for extreme agitated patients performed by EPs (board certified) (*n* = 251).

Reported prehospital sedation level by ambulance staff, according to EP (n, %)				
No sedation	16 (6.4%)			
Insufficient sedation	98 (39.0%)			
Sufficient sedation	113 (45.0%)			
Excessive sedation	24 (9.6%)			
	Most reported sedation used in ED when **NO** IV access is present*		Most reported sedation used in ED when IV access is present*	
	Responses, n (%)	IM as preferred route, n (%)	Responses, n (%)	IV as preferred route, n (%)
Midazolam	87 (34.7%)	63 (72.4%)	87 (34.7%)	85 (97.7%)
Droperidol	58 (23.1%)	58 (100.0%)	58 (23.1%)	54 (93.1%)
Droperidol and Midazolam	36 (14.3%)	31 (86.1%)**	50 (19.9%)	50 (100.0%)
Esketamine	26 (10.4%)	26 (100.0%)	21 (8.4%)	21 (100.0%)
Other sedative (combination)	44 (17.5%)	-	35 (13.9%)	-
Median dosage (IQR) per route				
	IM	IV	IN	
Midazolam	10.0 (5.0–10.0)	5.0 (5.0–7.5)	5.0 (5.0–10.0)	
Droperidol	10.0 (5.0–10.0)	5.0 (5.0–10.0)	-***	
Droperidol and Midazolam				
Droperidol	5.0 (5.0–10.0)	5.0 (5.0–5.0)	-***	
Midazolam	5.0 (5.0–5.0)	5.0 (5.0–5.0)	5.0 (5.0–5.0)	
Esketamine	150.0 (142.5–200.0)	70.0 (70.0–100.0)	-****	

IQR = Interquartile range; ED = Emergency Department; IM = intramuscular; IV = intravenous; IN = intranasal

* EPs were asked to indicate the sedative, dosage, and route of administration they would choose for an extremely agitated male patient weighing 70 kg;

** The other 13.9% respondents reported using IM droperidol followed by IN midazolam as their initial sedation strategy

*** Droperidol IN is not available

**** Esketamine IN was not selected by responders

### 3.3 Reasoning for sedative

The most frequently selected reason for choosing a specific initial sedative was adherence to the DSEP sedation pocket card for acute behavioural disturbances (43.0%), followed by the EP’s perceived effectiveness per agent (37.8%) and experience with specific agents during residency (30.3%). Factors most frequently influencing the choice of initial sedative included the severity of the patient’s agitation (68.9%), personal experience with different agents (58.2%), and prior agent used by emergency medical services (53.4%) (Table 3).

**Table 3:** Choice of specific initial sedative by EPs (board certified) (N = 251).

Reasons for Choosing a Specific Initial Sedative, *n* (%)*	
I follow the pocket card advice of the Dutch Society for EPs (DSEP)	108 (43.0%)
I believe this is the most effective sedative for extreme agitation	95 (37.8%)
This is how I was trained to sedate during my residency	76 (30.3%)
I follow protocols specific for the ED of the hospital where I currently work	68 (27.1%)
It is the “usual care” that we provide in the hospital where I work	48 (19.1%)
I follow hospital-specific guidelines of the institution where I currently work	19 (7.6%)
I have gained experience with this sedative abroad	18 (7.2%)
This is the only sedative available in the ED where I work	6 (2.4%)
I follow the national guideline on intoxications (NIV)	4 (1.6%)
Reported Factors Influencing the Choice of Sedative, *n* (%)	
Severity of the patient’s agitation	173 (68.9%)
Personal experience with different sedatives	146 (58.2%)
Sedation already administered by emergency medical services	134 (53.4%)
Patient’s medical history	130 (51.8%)
Underlying cause of agitation (e.g., intoxication, psychosis)	130 (51.8%)
Patient’s age	80 (31.9%)
Patient’s weight	60 (23.9%)
Experience of the ED team with different sedatives	57 (22.7%)
My choice is not influenced by external factors	15 (6.0%)
Patient’s sex	10 (4.0%)

ED = Emergency Department; *Participants were allowed to choose multiple options.

### 3.4 Experience per sedative

**Fig. 1:**
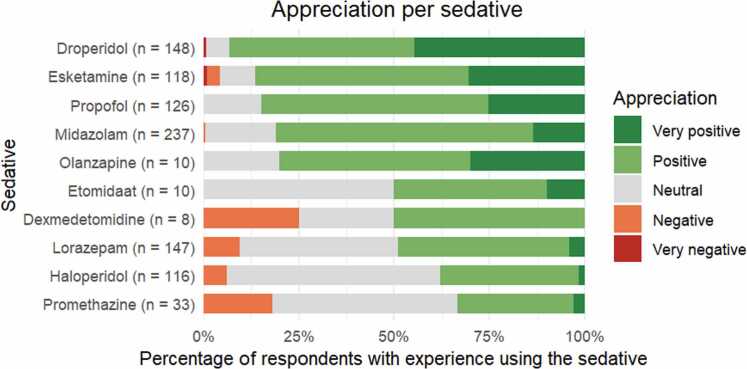
average appreciation score per sedative for EPs (Board certified).

Fig. 1 shows the average appreciation scores for individual sedatives, with droperidol, esketamine, propofol and midazolam receiving the most consistently positive evaluations; additionally, olanzapine was viewed positively, though only a small proportion of EPs reported experience with its use.

Among the most reported used agents, midazolam, droperidol, and esketamine, effectiveness and rapid onset were the most frequently cited positive attributes. Droperidol and esketamine were often associated with few side effects, whereas midazolam was more frequently linked to reports of adverse effects (Table 4).

**Table 4:** Top three reported experience per sedative by EPs (board certified) (N = 251)*.

Sedative	Reason 1	Reason 2	Reason 3
Droperidol	Effective agent 129 (87.2%)	Rapid onset 112 (75.7%)	Few side effects 87 (58.8%)
Esketamine	Effective agent 92 (78.0%)	Rapid onset 81 (68.6%)	Few side effects 47 (39.8%)
Etomidate	Rapid onset 7 (70.0%)	Effective agent 3 (30.0%)	Few side effects 1 (10.0%)
Propofol	Rapid onset 108 (85.7%)	Effective agent 90 (71.4%)	Many side effects 49 (38.9%)
Midazolam	Effective agent 176 (74.3%)	Rapid onset 139 (58.6%)	Many side effects 56 (23.6%)
Olanzapine	Effective agent 7 (70.0%)	Slow onset 5 (50.0%)	Rapid onset 2 (20.0%)
Dexmedetomidine	Limited efficacy 4 (50.0%)	Effective agent 3 (37.5%)	Few side effects 3 (37.5%)
Promethazine	Effective agent 11 (33.3%)	Slow onset 9 (27.3%)	Limited efficacy 8 (24.2%)
Lorazepam	Slow onset 76 (51.7%)	Effective agent 63 (42.9%)	Limited efficacy 27 (18.4%)
Haloperidol	Slow onset 43 (37.1%)	Effective agent 41 (35.3%)	Limited efficacy 35 (30.2%)

* Participants could select up to three predefined experiences per sedative from a list that included: “effective agent”, “rapid onset”, “slow onset”, “few side effects”, “many side effects”, “limited efficacy”, “not always in stock”, and “other”

### 3.5 Rescue medication

The majority (85.7%) preferred to repeat doses of their initial sedative agent before switching to another sedative after reaching a certain cumulative dose. Esketamine (32.7%) and propofol (27.5%) were the most reported rescue medications (Table 5).

**Table 5:** Rescue-sedative when initial sedative fails by EPs (board certified) (N = 251).

Management approach after failure of initial sedative, *n* (%)	
I escalate my initial sedative until successful, rarely switching.	18 (7.2%)
If the first dose of my initial sedative fails, I switch immediately.	18 (7.2%)
I repeat doses of my initial sedative but switch after a certain cumulative dose.	215 (85.7%)
Rescue-medication of choice, *n* (%)	
Esketamine	82 (32.7%)
Propofol	69 (27.5%)
Midazolam	38 (15.1%)
Droperidol	28 (11.2%)
Other sedative (combinations)	34 (13.5%)

### 3.6 EPs in training

Of the 166 eligible EPs in training, 60 responded (36.1%); after exclusion of 9 (5.4%) incomplete responses, 51 were analysed (30.7%). EPs in training had generally the same preferences for sedatives compared to board-certified EPs. However, EPs in training more frequently selected droperidol–midazolam than droperidol alone when IV access was not available compared to board certified EPs; No major differences between the groups were observed for IM sedative choices. Dosing was comparable between EPs and EPs in training: for IM administration, the median dose of midazolam and droperidol was 10 mg in both groups; for IV administration, the median dose midazolam and droperidol was 5.0 mg in both groups. EPs in training more often relied on DSEP recommendations (54.9% vs 43.0%) and local ED guidelines (33.3% vs 27.1%), whereas board-certified EPs more frequently selected sedatives based on personal experience (58.2% vs 29.4%) or on what they considered the most effective agent (37.8% vs 23.5%). Droperidol was considered most effective and safe by both groups. EPs in training more often switched to rescue medication after a single failed attempt with the initial sedative, whereas board-certified EPs tended to use multiple doses of the initial agent before switching. Both groups preferred esketamine as rescue therapy.

## 4 Discussion

This national survey is the first to systematically evaluate current sedation practices for extreme agitation among EPs. The findings mostly show variation within the generally recommended options, with midazolam, droperidol, and their combination most frequently used, consistent with national and international recommendations [26], [28]. Additionally, droperidol was the sedative with the highest appreciation, while midazolam remained the most frequently selected sedative in practice. Reported reasons for sedative choice varied, reflecting a combination of guideline adherence, clinical experience, and experience during emergency medicine training. Failed initial sedation was mostly managed by repeating the initial agent up to a certain cumulative dose before switching to a rescue sedative, mostly esketamine and propofol. The majority of respondents adapted their sedation strategy based on contextual factors such as the prehospital sedative used, the severity of agitation, and underlying aetiology, highlighting the complex and situational nature of pharmacological sedation in emergency care. Finally, differences in sedation preference between EPs in training and board-certified EPs suggest that trainees rely more on guidelines, whereas board-certified EPs rely more on personal experience.

Midazolam remains the most reported used sedative among Dutch EPs. It was frequently rated as both effective and fast-acting yet also associated with relatively frequent side effects. This aligns with existing literature, where midazolam is consistently recognised for its efficacy but also noted for its risk of respiratory depression and airway obstruction [9], [11], [12]. Prehospital sedation, in the Netherlands exclusively performed with midazolam, was perceived as insufficient by a large group of physicians [36]. This, too, reflects previous study results, concluding that midazolam often requires repeated dosing to achieve adequate sedation and has a slower onset compared to other agents, for instance droperidol [9], [11], [12], [37], [38]. In the Dutch prehospital context, however, limited transport times may restrict the opportunity for additional dosing. Moreover, suboptimal sedation may occur in older adults and in patients with chronic alcohol use, benzodiazepine dependence, or other substance use disorders (populations commonly seen in EDs), who may exhibit pharmacological tolerance or paradoxical agitation in response to midazolam administration [39].

Respondents were generally very positive about droperidol, frequently describing it as effective, fast-acting, and associated with few side effects. This is in line with the predominantly positive reports on droperidol within the emergency medicine literature [11], [12]; Although droperidol use in the United States largely disappeared from 2013 until 2019 following regulatory warnings about QTc prolongation and concurrent availability issues, this cessation reflected external restrictions rather than negative clinical experience [14], [15]. More recent evidence has challenged earlier safety concerns (particularly those regarding QTc prolongation) when droperidol is used at appropriate doses. Also, the respondents’ perceptions regarding side effects of droperidol aligned with these findings [11], [13], [16], [18].

When initial sedation is insufficient, most respondents first repeat doses of their initial sedative, switching to a rescue agent only after reaching a certain cumulative threshold. Many physicians reported preferring esketamine or propofol as rescue therapy when the initial sedation strategy proved insufficient. In a recent meta-analysis by deSouza et al. [9], ketamine emerged as one of the most effective agents for achieving rapid tranquillization in agitated patients [9]. However, its use is also associated with a relatively high incidence of adverse events, including emergence agitation, laryngospasm, and increased need for intubation [9], [40]. It should be noted that this survey specifically assessed esketamine, whereas deSouza et al. evaluated ketamine [9]. In contrast, evidence supporting the use of propofol specifically for extreme agitation is limited. Most available data on propofol in the emergency department relate to procedural sedation, which represents a clinically different scenario from the management of acute behavioural disturbance. In a recent systematic review by Khan et al. (2024) [41], examining procedural sedation in emergency settings, found that propofol was associated with higher rates of hypotension, apnoea, and hypoxia compared to esketamine, while esketamine was linked to more frequent reports of aspiration and agitation. Propofol has a narrow therapeutic window, with rapid progression to deep sedation and respiratory depression, and in clinical practice its use may require advanced airway management, including endotracheal intubation. As this study was based on survey responses, detailed information on whether airway management was performed when propofol was used was not collected. Consequently, whether propofol offers any meaningful advantage over esketamine as a rescue agent remains unclear [41].

This study has several limitations. First, the survey response rate was relatively low, which raises the possibility of non-response bias and may limit the representativeness of the findings. Second, there is a risk of response bias, as physicians who are more engaged with sedation or research may be more likely to participate. This could result in an overrepresentation of certain sedation strategies or more guideline-concordant responses, reducing the generalisability of the findings. Third, as the data are derived from a survey with a limited set of questions, they cannot fully capture the complexity of pharmacological sedation in extreme agitation, where decisions depend on dynamic factors such as the patient’s condition, underlying cause of agitation, staff availability and experience, and environmental safety. Lastly, as the survey was conducted exclusively in Dutch EDs, the generalizability to settings outside the Netherlands may be limited. However, the underlying challenges, balancing rapid sedation, safety, and variable staff experience, are universal, making the insights broadly applicable to international emergency care.

## 5 Conclusion

Dutch EPs primarily reported use a recommended set of sedatives based on national guidelines, but their approach to managing extreme agitation varies within that framework. Emergency physicians reported midazolam as the most frequently used first-line agent, although respondents generally rated droperidol as more effective and better tolerated. Given these perceptions and the available evidence supporting droperidol’s efficacy and safety, it may be reasonable to reconsider the prominent role of midazolam, particularly since prehospital midazolam sedation was often judged to be insufficient.

## CRediT authorship contribution statement

**J.J.J. OUWERKERK:** Writing – review & editing, Writing – original draft, Visualization, Software, Resources, Methodology, Investigation, Formal analysis, Data curation, Conceptualization. **N. KRAAIJVANGER:** Writing – review & editing, Writing – original draft, Visualization, Supervision, Resources, Methodology, Investigation, Funding acquisition, Conceptualization. **N.J.A van der WEE:** Writing – review & editing, Writing – original draft, Visualization, Supervision. **F.M.J. GRESNIGT:** Writing – review & editing, Writing – original draft, Visualization, Supervision, Resources, Methodology, Investigation, Conceptualization.

## Declaration of Competing Interest

The authors declare that they have no known competing financial interests or personal relationships that could have appeared to influence the work reported in this paper.

## Data Availability

Data will be made available on request.
