# Supplementary material for: National survey of Dutch emergency physicians on pharmacological sedation practices for extreme agitation
Source: Toxicol Rep. 2026 Mar 28;16:102246. doi: 10.1016/j.toxrep.2026.102246 (PMC13087722; doi:10.1016/j.toxrep.2026.102246)
Supplement: Supplementary file 1 — Supplementary material [file mmc1.docx]

***Appendix 1, Figure 2: DSEP sedation pocket card for acute behavioural disturbances translated from Dutch to English*** *
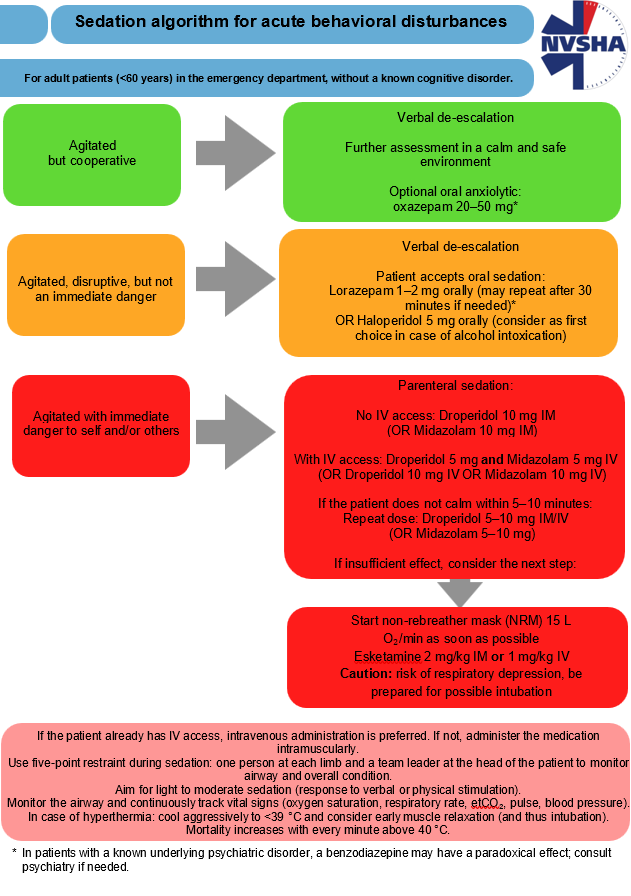
*
