# Supplementary material for: National survey of Dutch emergency physicians on pharmacological sedation practices for extreme agitation
Source: Toxicol Rep. 2026 Mar 28;16:102246. doi: 10.1016/j.toxrep.2026.102246 (PMC13087722; doi:10.1016/j.toxrep.2026.102246)
Supplement: Supplementary file 2 — Supplementary material [file mmc2.docx]

***Appendix 2: Survey send to all hospitals with an ED in the Netherlands***

Survey Pharmacological Sedation for Agitation in Dutch Emergency Departments

**Introduction**

Extreme agitation in the Emergency Department (ED) presents a significant challenge and poses risks to both patients and staff. Extreme agitation is defined as an acute behavioural disturbance requiring physical restraint and/or pharmacological intervention, as assessed by the ED nurse or ED physician.

**What is the purpose of this research?**

With this study, we aim to gain insight into the current practices in Dutch EDs regarding the pharmacological management of extreme agitation.

**How will the research be conducted?**

We kindly ask you to complete this online questionnaire, which will take approximately 5–10 minutes of your time.

**What are the potential benefits and drawbacks of participating in this research?**

By participating, you will contribute to scientific research on the pharmacological treatment of extreme agitation. You will not receive financial compensation for your participation.

**What will happen to my data?**

Participation in this research is anonymous. By completing the questionnaire, you give permission for the information you provide to be used and stored for scientific purposes. The research data will only be accessible to members of the research team, and only to the extent necessary for the conduct of this study. We are required to store the research data for 15 years. You cannot withdraw your consent for the use of your responses at a later time, as the data are anonymous and it is not possible to identify which responses are yours.

**We kindly request that you complete this survey only once.**

Thank you in advance for your time and effort.

Kind regards,

Joep Ouwerkerk, Emergency Medicine Resident (ANIOS) LUMC and PhD Candidate LUMC

**
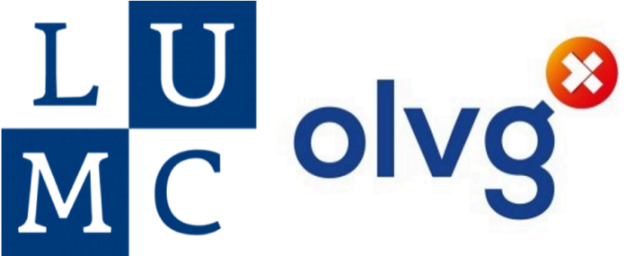
**On behalf of:

Dr Nicole Kraaijvanger, Emergency Physician LUMC

Dr Femke Gresnigt, Emergency Physician OLVG

Prof Dr Nic van der Wee, Psychiatrist LUMC

**1. Characteristics of an Emergency Physician and Emergency Physician in training**

1.1 Have you previously completed this questionnaire fully and clicked ‘Finish’?

- Yes *(the questionnaire would close if the option was selected)*
- No

1.2 What is your position in the hospital?

- Emergency Physician
- Emergency Medicine Resident (AIOS SEH)
- Other position (not listed above) *(the questionnaire would close if the option was selected)*

1.3 What is your gender?

- Male
- Female
- Other

1.4 What is your age?

- 20–29 years old
- 30–39 years old
- 40–49 years old
- 50–59 years old
- 60–69 years old
- ≥ 70 years old

**2. Work Experience of Emergency Physicians and Emergency Medicine Residents**

2.1 In which training region where you trained (or are you currently in training)?

- Region Northeast
- Region East North
- Region Utrecht
- Region Northwest North
- Region Leiden
- OOR Southwest North
- OOR Southeast North
- Training not completed in the Netherlands
- Other

2.2 In which hospital are you currently working?

*(Open text field)*

2.3 How many years of clinical experience do you have as a physician (since graduating from medical school)?

*(Numeric entry, in years)*

2.4 How many years of clinical experience do you have in the ED (including ANIOS, Residency, and as Emergency Physician)?

*(Numeric entry, in years)*

2.5 How many years of clinical experience do you have as an Emergency Physician?
Enter 0 if you are not yet an Emergency Physician

*(Numeric entry, in years)*

2.6 Have you gained clinical work experience in other medical specialties as a physician?
(Experience outside Emergency Medicine, excluding rotations during residency or internships)

- No, I have not worked in other medical specialties besides Emergency Medicine
- Yes, I have worked in other medical specialties besides Emergency Medicine *(if this option was selected, question 2.6.1 would appear)*

2.6.1 In which other medical specialties have you worked so far?
(Exclude specialties worked in during residency or internships)

- Anaesthesiology
- Cardiology
- ENT
- General Practice
- Gastroenterology
- Geriatrics
- Haematology
- Surgery
- Internal Medicine
- Intensive Care Medicine
- Neurology
- Obstetrics and Gynaecology
- Paediatrics
- Pulmonology
- Psychiatry
- Radiology
- Tropical Medicine
- Urology
- Other *(if this option was selected, question 2.6.1.1 would appear)*

2.6.1.1 Which other medical specialty have you worked in?

*(Open text field)*

2.7 How many years of clinical experience do you have as a physician abroad?
Enter 0 if you have no clinical experience abroad?

*(Numeric entry, in years) (If ≥ 1 year, then question 2.7.1 would appear)*

2.7.1

In which country or countries did you gain clinical experience as a physician?

*(Open multiline text field)*

**3. Hospital Characteristics of Emergency Physicians and Emergency Physicians in training**

3.1 How many patients visit your Emergency Department per year?

- Fewer than 15,000 patients
- 15,000–25,000 patients
- 25,000–35,000 patients
- More than 35,000 patients
- This information is unknown to me

3.2 Is there a 24/7 Emergency Physician present in the ED where you currently work?

- Yes, an Emergency Physician is present 24/7
- No, during certain times (e.g. at night) no Emergency Physician is present

**4. Prehospital sedation**

4.1 What is your assessment of the level of sedation when the ambulance has already initiated prehospital pharmacological sedation with midazolam?

- The patient is usually insufficiently sedated upon arrival.
- The patient is usually adequately sedated upon arrival.
- The patient is usually over-sedated upon arrival.
- Patients rarely arrive at our ED already sedated by EMS.

**5. First choice sedative**

5.1 What is your first choice regarding medication, dose, and route of administration when acutely sedating an extremely agitated patient (40-year-old male, 70 kg, without relevant medical history or contraindications) WITHOUT intravenous access?

If you combine two sedatives, you may indicate this in the second column. If you only use one sedative as first choice, leave the second column empty.

|  | First choice sedative | Optional combination sedative |
| --- | --- | --- |
| Type of sedative | --- | --- |
| Dose (mg) | --- | --- |
| Route of administration | --- | --- |

*Respondents could choose out of the following options for sedatives: dexmedetomidine, droperidol, etomidate, haloperidol, ketamine, lorazepam, midazolam, olanzapine, propofol, others.*

*Respondents could choose out of the following options for route of administration: intramuscular or intranasal.*

5.2 What is your first choice regarding medication, dose, and route of administration when acutely sedating an extremely agitated patient (40-year-old male, 70 kg, without relevant medical history or contraindications) WITH intravenous access?

If you combine two sedatives, you may indicate this in the second column. If you only use one sedative as first choice, leave the second column empty.

|  | First choice sedative | Optional combination sedative |
| --- | --- | --- |
| Type of sedative | --- | --- |
| Dose (mg) | --- | --- |
| Route of administration | --- | --- |

*Respondents could choose out of the following options for sedatives: dexmedetomidine, droperidol, etomidate, haloperidol, ketamine, lorazepam, midazolam, olanzapine, propofol, others.*

*Respondents could choose out of the following options for route of administration: intravenous, intramuscular or intranasal.*

5.3 What is the reason that you primarily chose this sedative or combination of sedatives in the previous question?

- I follow the ED-specific guidelines of the hospital where I currently work.
- I follow the hospital-specific protocols of the hospital where I currently work.
- I follow the NVSHA toxicology section pocket card.
- I follow the Dutch Internal Medicine (NIV) “Intoxications” guideline.
- In my opinion, this is the most effective option for sedation in extreme agitation.
- This is the sedation approach I was taught during training.
- This is the “usual care” provided in the hospital where I work.
- I gained experience with this medication abroad.
- This is the only sedative available in my ED.
- Other, namely… (if this option was selected, question 5.3.1 would appear)

5.3.1. If “Other, namely…”

*(Open text field)*

5.4 Are there specific factors that influence your choice of sedative?

- No, my choice of sedative is not influenced by external factors.
- Yes, the patient’s age.
- Yes, the patient’s weight.
- Yes, the patient’s gender.
- Yes, the patient’s medical history.
- Yes, the severity of agitation.
- Yes, the underlying cause of agitation (e.g. intoxication, psychosis).
- Yes, my personal experience with different agents.
- Yes, personal experience of the ED team with different agents.
- Yes, prior sedation administered by EMS before arrival.
- Other, namely… (if this option was selected, question 5.3.1 would appear)

5.4.1. If “Other, namely…”

*(Open text field)*

**6. Experience per sedative**

6.1 Which agents have you personally used to sedate patients with extreme agitation?

Extreme agitation is defined as an acute behavioural disturbance requiring physical restraint and/or pharmacological intervention, as assessed by the ED nurse or ED physician.

- Dexmedetomidine
- Droperidol
- Etomidate
- Haloperidol
- Ketamine
- Lorazepam
- Midazolam
- Olanzapine
- Promethazine
- Propofol
- Other

*For each sedative selected, the following questions will appear.*

6.1.x What is your experience with xxxxx?

- Very positive
- Positive
- Neutral
- Negative
- Very negative

6.1.x What is the reason for your answer regarding your experience with dexmedetomidine?

- Rapid onset
- Effective agent
- Few side effects
- Slow onset
- Limited effectiveness
- Many side effects
- Not always in stock
- Other *(if other was selected, option 6.1.x.x would appear)*

6.1.x.x.

*(Open text field)*

**7. Rescue medication**

7.1 If sedation with your first-choice sedative (or combination of sedatives) is not sufficiently effective, what is your next step?

- I continue administering higher doses of my first-choice sedative until sedation is achieved; I almost never switch to another sedative.
- If the first dose of my first-choice sedative is not sufficiently effective, I immediately switch to another agent.
- I administer additional doses of my first-choice sedative; however, after a certain total dose, I will switch to another agent.

7.2 At approximately which total dose of your first-choice sedative would you switch to another sedative if agitation does not reduce?

We understand every patient is different, but please provide a general estimate for a 40-year-old male, 70 kg, without relevant medical history or contraindications.

*(Numeric entry, mg)*

7.3 What is your first choice for rescue medication (medication, initial dose, and route of administration) when acutely sedating an extremely agitated patient (40-year-old male, 70 kg, without relevant medical history or contraindications) if your first-choice sedative(s) are ineffective after adequate dosing?

If you combine two sedatives, indicate this in the second column. If you only use one sedative as first choice, leave the second column empty.

|  | First choice sedative | Optional combination sedative |
| --- | --- | --- |
| Type of sedative | --- | --- |
| Dose (mg) | --- | --- |
| Route of administration | --- | --- |

*Respondents could choose out of the following options for sedatives: dexmedetomidine, droperidol, etomidate, haloperidol, ketamine, lorazepam, midazolam, olanzapine, propofol, others.*

*Respondents could choose out of the following options for route of administration: intravenous, intramuscular or intranasal.*

**Final statement when finalizing the survey**

Thank you for participating in this study. We greatly appreciate your time and effort.
The questionnaire will be completed once you click “Finish”.
