# Supplementary material for: National survey of Dutch emergency physicians on pharmacological sedation practices for extreme agitation
Source: Toxicol Rep. 2026 Mar 28;16:102246. doi: 10.1016/j.toxrep.2026.102246 (PMC13087722; doi:10.1016/j.toxrep.2026.102246)
Supplement: Supplementary file 3 — Supplementary material [file mmc3.docx]

***Appendix 3, table 6: tables with results for EP-training***

| Table 6. Characteristics of participating EPs (in training) (*n* = 51) | |
| --- | --- |
| Sex (*n*, %) |  |
| Male | 16 (30.2%) |
| Female | 35 (68.6%) |
| Other | 0 (0.0%) |
| Age (years) |  |
| Between 20-29 | 7 (13.2%) |
| Between 30-39 | 41 (80.4%) |
| Between 40-49 | 3 (5.9%) |
| Between 50-59 | 0 (0.0%) |
| Between 60-69 | 0 (0.0%) |
| Clinical experience |  |
| As physician in general (years, SD) | 5.0 (5.0–7.0) |
| As physician working in the ED (years, SD) | 4.0 (3.2–5.0) |
| Hospital characteristics |  |
| 24/7 staffing with EPs | 44 (86.3%) |
| Geographic classification, *n* (%) |  |
| Urban | 51 (100.0%) |
| Intermediate | 0 (0.0%) |
| Rural | 0 (0.0%) |
| Trauma level, *n* (%) |  |
| Level 1 | 13 (25.5%) |
| Level 2 | 38 (74.5%) |
| Level 3 | 0 (0.0%) |
| Academic status | 7 (13.7%) |
| SD = Standard deviation; EP = Emergency Physician; ED = Emergency Department | |
