# Supplementary material for: National survey of Dutch emergency physicians on pharmacological sedation practices for extreme agitation
Source: Toxicol Rep. 2026 Mar 28;16:102246. doi: 10.1016/j.toxrep.2026.102246 (PMC13087722; doi:10.1016/j.toxrep.2026.102246)
Supplement: Supplementary file 4 — Supplementary material [file mmc4.docx]

***Appendix 4, table 7: tables with results for EP-training***

| **Table 7. Characteristics of initial pharmacological sedation for extreme agitated patients performed by EPs in training (*n* = 51)** | | | | |
| --- | --- | --- | --- | --- |
| Reported prehospital sedation level by ambulance staff, according to EP (n, %) | | | | |
| No sedation | 5 (9.8%) |  |  |  |
| Insufficient sedation | 18 (35.3%) |  |  |  |
| Sufficient sedation | 18 (35.3%) |  |  |  |
| Excessive sedation | 10 (19.6%) |  |  |  |
|  |  |  |  |  |
|  | Most common sedation used in ED when **NO** IV access is present* | | Most common sedation used in ED when IV access is present* | |
|  | Responses, n (%) | IM as preferred route, n (%) | Responses, n (%) | IV as preferred route, n (%) |
| Midazolam | 21 (41.2%) | 18 (85.7%) | 18 (35.3%) | 18 (100.0%) |
| Droperidol | 10 (19.6%) | 10 (100.0%) | 16 (31.4%) | 14 (87.5%) |
| Droperidol and Midazolam | 16 (31.4%) | 15 (93.8%)** | 15 (29.4%) | 15 (100.0%) |
| Esketamine | 1 (2.0%) | 1 (100.0%) | 0 (0.0%) | 0 (0.0%) |
| Other sedative (combination) | 3 (5.9%) | - | 2 (3.9%) | - |
|  |  |  |  |  |
| Median dosage (IQR) per route |  |  |  |  |
|  | IM | IV | IN |  |
| Midazolam | 10.0 (10.0–10.0) | 5.0 (5.0–8.8) | 5.0 (5.0–7.5) |  |
| Droperidol | 10.0 (7.5–10.0) | 5.0 (5.0–8.8) | -*** |  |
| Droperidol and Midazolam |  |  |  |  |
| Droperidol | 7.5 (5.0–10.0) | 5.0 (5.0–5.0) | -*** |  |
| Midazolam | 5.0 (5.0–10.0) | 5.0 (5.0–5.0) | 5.0 (5.0–5.0) |  |
| Esketamine | 150.0 (150.0–150.0) | -**** | -**** |  |
| IQR = Interquartile range; ED = Emergency Department; IM = intramuscular; IV = intravenous; IN = intranasal * EPs were asked to indicate the sedative, dosage, and route of administration they would choose for an extremely agitated male patient weighing 70 kg;  ** The other 6.2% respondents reported using IM droperidol followed by IN midazolam as their initial sedation strategy  *** Droperidol IN is not available  **** Esketamine IN was not selected by responders | | | | |
