# Supplementary material for: National survey of Dutch emergency physicians on pharmacological sedation practices for extreme agitation
Source: Toxicol Rep. 2026 Mar 28;16:102246. doi: 10.1016/j.toxrep.2026.102246 (PMC13087722; doi:10.1016/j.toxrep.2026.102246)
Supplement: Supplementary file 5 — Supplementary material [file mmc5.docx]

***Appendix 5, table 8: tables with results for EP-training***

| Table 8. Choice of specific initial sedative by EPs (in training) (N = 51) | |
| --- | --- |
| Reasons for Choosing a Specific Initial Sedative, *n* (%)* |  |
| I follow the pocket card advice of the Dutch Society for EPs (DSEP) | 28 (54.9%) |
| This is how I was trained to sedate during my residency | 22 (43.1%) |
| I follow protocols specific for the ED of the hospital where I currently work | 17 (33.3%) |
| I believe this is the most effective sedative for extreme agitation | 12 (23.5%) |
| It is the “usual care” that we provide in the hospital where I work | 11 (21.6%) |
| I follow hospital-specific guidelines of the institution where I currently work | 6 (11.8%) |
| I follow the national guideline on intoxications (NIV) | 2 (3.9%) |
| I have gained experience with this sedative abroad | 2 (3.9%) |
| This is the only sedative available in the ED where I work | 1 (2.0%) |
| Reported Factors Influencing the Choice of Sedative, *n* (%) |  |
| Severity of the patient’s agitation | 32 (62.7%) |
| Personal experience with different sedatives | 15 (29.4%) |
| Sedation already administered by emergency medical services | 31 (60.8%) |
| Patient’s medical history | 21 (41.2%) |
| Underlying cause of agitation (e.g., intoxication, psychosis) | 24 (47.1%) |
| Patient’s age | 17 (33.3%) |
| Patient’s weight | 15 (29.4%) |
| Experience of the ED team with different sedatives | 15 (29.4%) |
| My choice is not influenced by external factors | 2 (3.9%) |
| Patient’s sex | 3 (5.9%) |
| ED = Emergency Department; *Participants were allowed to choose multiple options. | |
