# Supplementary material for: National survey of Dutch emergency physicians on pharmacological sedation practices for extreme agitation
Source: Toxicol Rep. 2026 Mar 28;16:102246. doi: 10.1016/j.toxrep.2026.102246 (PMC13087722; doi:10.1016/j.toxrep.2026.102246)

***Appendix 6, figure 3: tables with results for EP-training***

Figure 3: average appreciation score per sedative for EPs (in training)


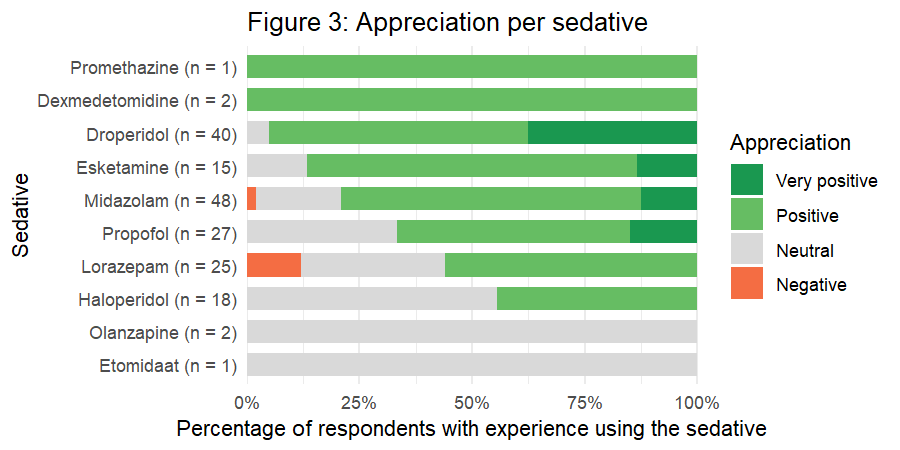

Supplement: Supplementary file 6 — Supplementary material [file mmc6.docx]
