# Supplementary material for: National survey of Dutch emergency physicians on pharmacological sedation practices for extreme agitation
Source: Toxicol Rep. 2026 Mar 28;16:102246. doi: 10.1016/j.toxrep.2026.102246 (PMC13087722; doi:10.1016/j.toxrep.2026.102246)
Supplement: Supplementary file 7 — Supplementary material [file mmc7.docx]

***Appendix 7, table 9: tables with results for EP-training***

| Table 9. Top three reported experience per sedative by EPs (in training) (N = 51)* | | | |
| --- | --- | --- | --- |
| Sedative | Reason 1 | Reason 2 | Reason 3 |
| Droperidol | Effective agent   33 (82.5%) | Rapid onset  22 (55.0%) | Few side effects  20 (50.0%) |
| Esketamine | Effective agent  11 (73.3%) | Rapid onset  10 (66.7%) | Few side effects  6 (40.0%) |
| Etomidate | Rapid onset  1 (100.0%) | - | - |
| Propofol | Rapid onset  21 (77.8%) | Effective agent  21 (77.8%) | Many side effects  11 (40.7%) |
| Midazolam | Effective agent  34 (70.8%) | Rapid onset  28 (58.3%) | Many side effects  11 (22.9%) |
| Olanzapine | Many side effects  1 (50.0%) | Not available in ED  1 (50.0%) | - |
| Dexmedetomidine | Effective agent  2 (100.0%) | - | - |
| Promethazine | Rapid onset  1 (100.0%) | Not available in ED  1 (100.0%) | - |
| Lorazepam | Effective agent  14 (56.0%) | Slow onset  12 (48.0%) | Rapid onset  5 (20.0%) |
| Haloperidol | Limited Efficacy  10 (55.6%) | Slow onset  6 (33.3%) | Effective agent 5 (27.8%) |
| *Participants could select up to three predefined experiences per sedative from a list that included: “effective agent”, “rapid onset”, “slow onset”, “few side effects”, “many side effects”, “limited efficacy”, “not always in stock”, and “other” | | | |
