# Supplementary material for: National survey of Dutch emergency physicians on pharmacological sedation practices for extreme agitation
Source: Toxicol Rep. 2026 Mar 28;16:102246. doi: 10.1016/j.toxrep.2026.102246 (PMC13087722; doi:10.1016/j.toxrep.2026.102246)
Supplement: Supplementary file 8 — Supplementary material [file mmc8.docx]

***Appendix 8, table 10: tables with results for EP-training***

| Table 10. Rescue-sedative when initial sedative fails by EPs (in training) (N = 51) | |
| --- | --- |
| Management approach after failure of initial sedative, *n* (%) |  |
| I continue administering higher doses of my initial sedative until sedation is successful; I (almost) never switch to another agent. | 3 (5.9%) |
| If the first dose of my initial sedative is not sufficiently effective, I immediately switch to another agent. | 9 (17.6%) |
| I repeat multiple doses of my first-choice sedative; however, I will switch to another agent after a certain cumulative dose. | 39 (76.5%) |
| Rescue-medication of choice, *n* (%) |  |
| Esketamine | 18 (35.3%) |
| Propofol | 8 (15.7%) |
| Midazolam | 11 (21.6%) |
| Droperidol | 9 (17.6%) |
| Other sedative (combinations) | 5 (9.8%) |
|  | |
